# Prediction of Protein-Protein Interactions Based on Domain

**DOI:** 10.1155/2019/5238406

**Published:** 2019-08-21

**Authors:** Xue Li, Lifeng Yang, Xiaopan Zhang, Xiong Jiao

**Affiliations:** ^1^College of Biomedical Engineering, Taiyuan University of Technology, Taiyuan 030600, China; ^2^College of Information and Computer, Taiyuan University of Technology, Taiyuan 030600, China; ^3^Department of MRI, The First Affiliated Hospital of Zhengzhou University, Zhengzhou 450052, China

## Abstract

Protein-protein interactions (PPIs) play a crucial role in various biological processes. To better comprehend the pathogenesis and treatments of various diseases, it is necessary to learn the detail of these interactions. However, the current experimental method still has many false-positive and false-negative problems. Computational prediction of protein-protein interaction has become a more important prediction method which can overcome the obstacles of the experimental method. In this work, we proposed a novel computational domain-based method for PPI prediction, and an SVM model for the prediction was built based on the physicochemical property of the domain. The outcomes of SVM and the domain-domain score were used to construct the prediction model for protein-protein interaction. The predicted results demonstrated the domain-based research can enhance the ability to predict protein interactions.

## 1. Introduction

Protein commonly consists of one or more submolecule parts, which are termed as domain. Domain is a structural or functional module of protein, and it is usually evolutionarily conserved units. Differential association of domains provides a way to create new functions for organisms [1]. The interactions between domains can help locate a protein at a specific subcellular site, which recognize protein posttranslational modification or participate in signal transduction. The interactions can also regulate the enzymatic activity, vigor, and substrate specificity [2]. Recently, many comprehensive studies about domain have been conducted. For example, PDZ domain, which was found in various proteins, including protein tyrosine phosphatase and nitric oxide synthase, plays an important role in regulating protein-protein interactions, protein targets, and protein complex formations [3]. The PB1 domain exists in many signaling proteins involved in the multiple signaling pathway, including the mitogen-activated protein kinase pathway [4] and cellular polarity pathways [5]. Proteins containing the PB1 domain have a close relationship to the occurrence of cancer, such as breast cancer and lung cancer. More and more findings indicate that abnormalities in the domain can lead to various diseases. Therefore, it holds an important practical significance for the domain-based drug design and disease treatment in clinical research, such as arteriosclerosis and cancer. Domain-based studies might help to understand the molecular mechanisms of human diseases, to develop appropriate disease models, and to provide tools for diagnosis.

Domain-based prediction has provided a new perspective for the study of protein-protein interactions (PPIs). PPIs play a crucial role in biological processes, including immune response, signal transduction, and the occurrence and development of disease. Usually, there are two methods predicting protein-protein interactions, experimental method and computational method. Experimental techniques identifying protein-protein interactions are the earliest research methods, including yeast two-hybrid (Y2H) [6], tandem affinity purification (TAP) [7], co-immunoprecipitation (Co-IP) [8], and other techniques. However, high- and low-throughput experimental techniques have some constraints on manpower and material, and experimental results often have high false positives and false negatives. Thus, computational methods have been developed for PPI prediction. The classification of computational method is mainly based on its different features. The commonly used features are protein sequence, protein evolutionary, three-dimensional structure, and domain information. Currently, sequence-based methods have achieved some good prediction results [9–18]. You et al. [19] considered the sequence order and dipeptide information of the protein primary sequence and proposed a matrix-based representation of protein sequence, which is used as the input information of an SVM. However, the sequence-based approaches only use the sequential information, and the 3D structure information was ignored. It is generally believed that protein interactions are mediated by some their specific domain interactions [20], so the domain-based method is widely used in recent years.

Wojcik and Schachter have developed an interacted domain pair profile method to predict protein-protein interactions. They applied their method to predict an interaction map of *Escherichia coli* [21]. Kim et al. have proposed a statistical scoring system, based on the interacting domain pairs from InterPro, to measure the interaction probability between domains and to represent protein-protein interactions [22]. Hayashida et al. have used conditional random field to predict PPIs based on mutual information between residues of domain-domain interactions [23]. Kamada et al. have used domain features with support vector regression (SVR) and relevance vector machine (RVR) to predict the strengths of PPIs [24]. Singhal and Resat have applied the InterDom (the interacting domain database) domain-domain interaction scores as the feature information. They developed a multiparameter optimization method—DomainGA—which used the obtained score to predict the interactions between proteins [25]. Zhang et al. have also used the domain-domain interaction scores. His method used DDI confidence probabilities to calculate the confidence probability of the predicted PPI [26].

Currently, the features that domain-based methods used just contain the domain co-occurrence relationships or the proportion of an important domain. The domain information is not completely considered. The domain interactions, which are crucial to the understanding of biomolecule interactions, also provide a global view of the protein-protein interaction network. In order to effectively utilize the information of the domain, we proposed a new domain-based method to predict protein-protein interactions.

In this paper, we both considered the physicochemical property of domain and the domain-domain interaction score. The physicochemical property of domain was used as the SVM feature to construct the DDI prediction model. Finally, the DDI prediction model is combined with the domain-domain interaction score to construct the PPI prediction model.

## 2. Materials and Methods

### 2.1. Proposed Method

**Figure 1:**
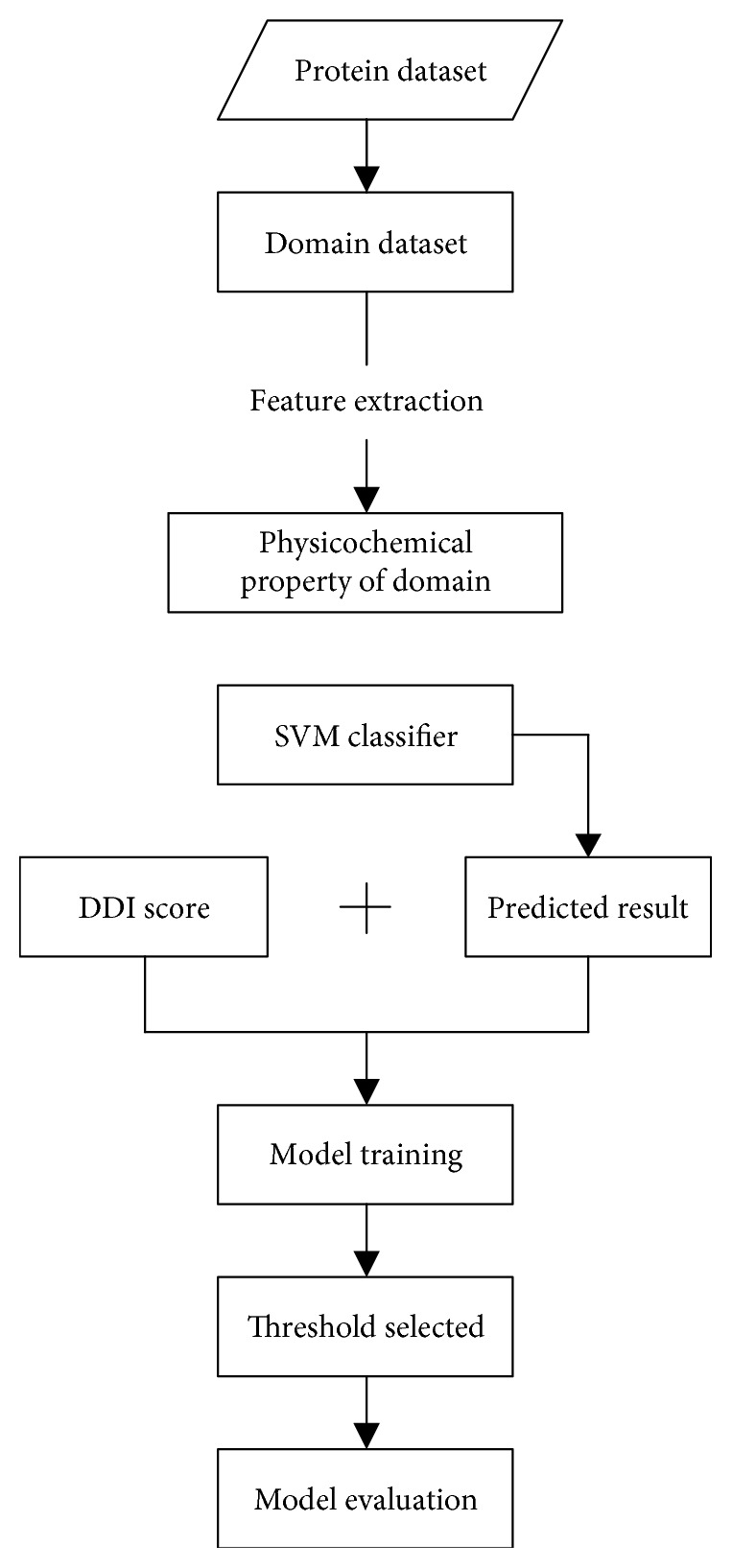
Flow chart of the method.

The flow chart of our method is given in Figure 1.

### 2.2. Datasets

#### 2.2.1. Protein Dataset

The positive protein-protein interaction data were collected from the interacting adhesome protein-protein. It can be obtained on the website of The Adhesome: A Focal Adhesion Network (http://www.adhesome.org/) [27, 28]. Xiao-Yong et al.'s noninteraction dataset, where any protein pair does not have sequence identity higher than 25% [29], was used for obtaining the negative PPI data. Pan's dataset was commonly used in protein-protein interaction studies [30, 31].

#### 2.2.2. Domain Dataset

We used the protein database mentioned above as our source database to extract the domain of its protein. The domains of protein and sequence information of these domains were obtained from the Pfam database (version 32.0 http://pfam.xfam.org/). We constructed the corresponding domain-domain pairs. Meanwhile, interacting and noninteracting domain pairs were chosen in the InterDom database (interacting domains http://interdom.lit.ofg.sg/) and 3did database (https://3did.irbbarcelona.org/index.php). The InterDom database had a set of confidence scores of DDIs which used 1.5 as the cutoff of false-positive and nonfalse-positive prediction [32]. The interacting domain-domain was selected where the Interdom score is greater than 1.5. Noninteracting DDIs are not available in the two domain level databases which we used above.

The positive protein dataset contained 427 positive PPI, and we constructed 1040 positive DDI from it. There were 403 noninteracting protein pairs, in the negative protein dataset, and we constructed 1040 negative DDI from it. The Interdom score was used in our protein-protein predicting model. But the domain-domain interaction score was not available for the negative domain dataset. So, we set up a score as a background noise to the negative DDI, which was chosen from the Interdom score in the positive domain dataset. 1040 values were ranged from small to large, and the 20% position value of 1.74 was selected.

### 2.3. Feature Extraction

The physicochemical property of domain pairs was used as features of our method. The domain and the corresponding sequence information were downloaded from the Pfam database. According to the sequence information of the domain, the physicochemical property of the domain can be obtained with the online tools ProtParam (https://web.expasy.org/protparam/) and ProtComp (https://www.expasy.org/). ProtParam can calculate various physicochemical parameters for a given protein [33]. ProtComp can predict the subcellular localization of animal/fungi proteins (version 9.0 http://www.softberry.com/berry.phtml?group=programs&subgroup=proloc&topic=protcompan). Thelocation of a protein in a cell has a close relationship to its biological function [34]. The detailed calculated parameter for ProtParam is listed in Table 1.

ProtComp calculated the weight of each position from ten positions and chose the most accurate one. To numerically represent the feature of the domain-domain pairs, ten domain location's information was encoded into numbers as shown in Table 2.

In order to reduce the interference of correlation factors, we carried out a correlation analysis for these features. Finally, ten meaningful physicochemical property features were picked out. They were amino acid numbers, theoretical pI, total number of negatively charged residues, total number of positively charged residues, total number of atoms, Ext. coefficient 1, instability index, aliphatic index, grand average of hydropathicity, and the domain location.

To reduce the impact of large differences in values between various features on results, we did normalized processing for these features according to Mapminmax function. Equation (1) is defined as follows:

(1) $$\begin{array}{c} y=\frac{\left(y_{\max}-y_{\min}\right)\;*\;\left(x-x_{\min}\right)}{\left(x_{\max}-x_{\min}\right)+y_{\min}}. \end{array}$$

The specific value of twenty physicochemical properties for domain was listed in Supplementary Tables [Supplementary-material supplementary-material-1] and [Supplementary-material supplementary-material-1]. Finally, the feature of the DDI was a 20-dimensional eigenvector.

### 2.4. Classification

There are numerous machine-learning techniques for predicting protein-protein interactions. Support vector machine (SVM) is the usual technique for classification and regression [35, 36]. In recent years, it has been widely used in bioinformatic researches and has made outstanding performances [30, 31, 37–41]. In this paper, SVM was used to design the classifier. The domain pairs class label was set +1 for interacting pairs and 0 for noninteracting pairs. The kernel function plays an important role in nonlinear classification. In this paper, the RBF kernel was chosen as the kernel function. The optimal parameters *c* and *g* were 9.1896 and 3.0314, which were optimized by the grid search method for SVM classifiers. The fivefold cross-validation method indicates that the data are randomly divided into five equal parts. One part is used as a testing set in turn, and the other four parts are used as a training test. It can effectively prevent the overfitting problem. At the same time, our results have been counted at least five times until the results are relatively stable.

The software libsvm 3.23 (http://www.csie.ntu.edu.tw/∼cjlin/libsvm/) was employed in this work.

### 2.5. Assessment of Prediction System

In order to evaluate the prediction performance of our approach, the following six measurements: accuracy (Acc), sensitivity (SN), specificity (SPE), precision (Pre), Matthews correlation coefficient (MCC), and *F*_1_ score values were used. Their mathematical description is defined as follows:

(2) $$\begin{array}{c} \text{Acc}=\frac{\text{TP}+\text{TN}}{\text{TP}+\text{FP}+\text{TN}+\text{FN}},\\ \text{SN}=\frac{\text{TP}}{\text{TP}+\text{FN}},\\ \text{SPE}=\frac{\text{TN}}{\text{TN}+\text{FP}},\\ \text{Pre}=\frac{\text{TP}}{\text{TP}+\text{FP}},\\ \text{MCC}=\frac{\text{TP}\times \text{TN}-\text{FP}\times \text{FN}}{\sqrt{\left(\text{TP}+\text{FN}\right)\times \left(\text{TN}+\text{FP}\right)\times \left(\text{TP}+\text{FP}\right)\times \left(\text{TN}+\text{FN}\right)}},\\ F_{1}=\frac{2\text{TP}}{2\text{TP}+\text{FP}+\text{FN}}, \end{array}$$

where TP (the true positive value) is the number of interactions predicted correctly; TN (the true negative value) is the number of noninteraction pairs predicted correctly; and FN (the false negative value) and FP (the false positive value) are the number of interactions incorrectly predicted as noninteractions and noninteracting proteins incorrectly as interactions.

## 3. Results and Discussion

This section is divided into four parts: the first part is the intermediate result of the prediction of the domain-domain interaction, the second part is the result of protein prediction, the third part is the comparison of different methods, and the last part is the limitations of our model.

### 3.1. Results of DDIs

We used the physicochemical property of domain to build the SVM prediction model. To evaluate the robustness of our method and to reduce impact of data independence, fivefold cross validation was used to ensure the reliability of the results. The SVM calculation was run five times. The result of domain-domain interaction prediction is shown in Table 3.

From Table 3, we can see that the DDI prediction model achieved an acceptable performance. The highest prediction accuracy was 95.24%. The average prediction accuracy was 94.69%. Two indicators, the *F*_1_ and MCC, can better evaluate the overall performance of the classifier. The average value of *F*_1_ was 94.54%, and the MCC was 89.39%. These results show that the domain's physicochemical properties are effective feature information for domain-domain interaction.

### 3.2. Results of PPIs

The domain-domain interaction score in the Interdom database and DDI predicted label results were used to build a protein-protein prediction model. In order to reduce the numerical difference between the domain-domain score, the value was obtained by the following algorithm:

(3) $$\begin{array}{c} \;D_{mn}=\frac{\log\;\lambda_{mn}}{\log\;S_{\text{max}}}. \end{array}$$

In which *λ*_*mn*_ represented the Interdom score of *m* domain and *n* domain pair and the *S*_max_ represented the maximum score of domain-domain in our database.  *P*_*ij*_ represented the DDI-predicted label results, which was a probability score that the interacting domain-domain we predicted to the total theoretical domain pairs in a protein pair.  *P*_*ij*_ was defined by using the following equation:

(4) $$\begin{array}{c} P_{ij}=\frac{\text{Num\_predicted}}{\text{Num\_DDI}}. \end{array}$$

Num_predicted was the number of predicted domain pairs with our model for one certain protein pair. Num_DDI was the theoretical number of all domain-domain pairs in the same protein pair.

In this section, we assumed that domain-domain interactions were independent [42]. We estimated the probability of each PPI by the following equation:

(5) $$\begin{array}{c} \;P_{mn}=A\;*\;D_{mn}+B\;*\;P_{ij}. \end{array}$$

Grid algorithm *N* *∗* *N* is used to find the optimal parameters *A* and *B*. We set the value of *N* from 0 to 0.6 by 0.1. A total of 49 uniform lattices trained the protein sets. In order to evaluate the results of the training, we set ten thresholds from 0.1 to 0.55, with an interval of 0.05. The values of TP, TN, FP, FN, the false negative rate (fn), the false positive rate (fp), ACC, and SN were calculated. These evaluation indexes are described in detail in Section 2.5. The specific algorithm for fn and fp was as follows:

(6) $$\begin{array}{c} \text{fn}=\frac{\text{FN}}{\text{FN}+\text{TP}},\\ \text{fp}=\frac{\text{FP}}{\text{FP}+\text{TN}}. \end{array}$$

The results of protein-protein interacting possibility were compared by the accuracy and ROC curves with AUC scores. Finally, the parameters A and B with high accuracy and large ACU area were selected. The final equation was as follows:

(7) $$\begin{array}{c} P_{mn}=0.5\;*\;D_{mn}+0.5\;*\;P_{ij}. \end{array}$$

**Figure 2:**
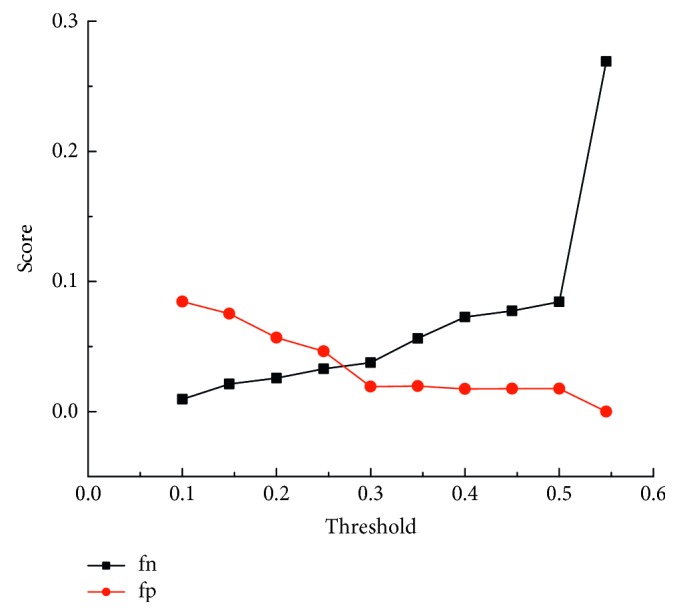
fn and fp of the predicted result.

In order to select the optimal threshold, we used formula (7) to train the parameter for the protein-protein dataset. The result is shown in Figure 2.

The *X*-axis represented different thresholds, and the *Y*-axis represented the values of fn and fp. The suitable threshold was determined according to two principles: (1) fn and fp should be as small as possible and (2) fn and fp should be as equal as possible. Therefore, we chose 0.26 as the optimal threshold. To obtain a more accurate threshold, we calculated the protein training results of three thresholds that are 0.25, 0.26, and 0.27. We also calculated the AUC (the area under the ROC curve). The results showed the same result in Table 4, so we chose 0.26 as the optimal threshold.

### 3.3. Comparison with Different Prediction Methods

To demonstrate the prediction performance, we compared our method with other SVM-based methods. In order to compare more accurately, we chose the different studies which not only used Pan's database but also used SVM classifier. The results are shown in Table 5.

As shown in Table 5, among different methods, the performance of our method achieved the best result. This suggests that our method based on domain to predict protein-protein interactions is relatively successful.

### 3.4. Limitations of Our Model

Although the accuracy of our method is acceptable, there are still some limitations for our model to be used widely. For example, the number of our dataset and the physiochemical property are small, and in future work, we plan to test our model on a bigger dataset with more features. For our approach, independent software and online tools development work are still in progress.

## 4. Conclusions

In this paper, we proposed a new domain-based method to predict protein-protein interaction. We used the domain's physicochemical property and interaction score to construct the protein interaction-predicting model. The predicted result, which achieved a good performance, indicates that our method is relatively successful. The physicochemical property of the domain as features for PPI prediction is of great significance. Applying our approach to large dataset and finding more effective feature information for predicting PPI will be part of our future work. Furthermore, our methods can be used for the prediction of new PPIs, and the result could provide some reference significance for dealing with related bioinformatics problems.

## Figures and Tables

**Table 1:** Parameters for ProtParam.

Molecular weight	Aliphatic index
Atom composition	Instability index
Theoretical pI	Ext. coefficient (1)
Number of amino acids	Ext. coefficient (2)
Amino acid composition (20)	Estimated half-life
Total number of negatively charged residues (Asp + Glu)	Extinction coefficients
Total number of positively charged residues (Arg + Lys)	Grand average of hydropathicity (gravy)

**Table 2:** Encoded information for the location.

Location	Code
Nuclear	1
Plasma membrane	2
Extracellular	3
Cytoplasmic	4
Mitochondrial	5
Endoplasmic reticulum	6
Peroxisomal	7
Lysosomal	8
Golgi	9
Vacuolar	10

**Table 3:** Performances of result for five-time predictions with SVM.

Time	Acc (%)	SN (%)	SPE (%)	Pre (%)	*F *_1_ (%)	MCC (%)
1	94.62	95.19	94.04	94.11	94.65	89.24
2	94.13	94.23	94.04	94.05	94.14	88.27
3	94.95	95.00	94.90	94.91	94.95	89.90
4	95.24	95.38	95.10	95.11	95.25	90.48
5	94.52	94.42	94.62	94.61	94.51	89.04

**Table 4:** Prediction results based on three thresholds.

Threshold	TP	TN	FP	FN	Acc (%)	SN (%)	SPE (%)	Pre (%)	MCC (%)	*F*_1_ (%)	AUC (%)
0.25	413	383	20	14	95.90	96.72	95.04	95.38	91.81	96.00	91.92
0.26	413	383	20	14	95.90	96.72	95.04	95.38	91.81	96.00	91.92
0.27	413	383	20	14	95.90	96.72	95.04	95.38	91.81	96.00	91.92

**Table 5:** Results of comparison with different methods.

Method	Acc (%)	SN (%)	Pre (%)	MCC (%)
This paper	95.9	96.72	95.04	91.81
Zhang^1^	82.11	80.4	84.73	80.07
SP-SVM^2^	70	66	72	—
LDA-SVM^3^	69	63	72	—
PSEAAC_SVM^4^	68	63	70	—
Yunus^5^	93.45	89.29	89.84	85.71

^1^The result was taken from Table 4 of Zhang et al.'s literature [26]. ^2–4^These results were taken from in Table 5 of Xiao-Yong et al.'s literature [29]. ^5^This was from Table 7 of Göktepe and Kodaz's literature [31].

## Data Availability

The physicochemical property of the domain and corresponding protein data used to support the findings of this study are included within the supplementary information files (Supplementary Tables [Supplementary-material supplementary-material-1] and [Supplementary-material supplementary-material-1]).

## Supplementary Materials

Supplementary Materials Supplementary Table S1: 1040 positive DDI. Supplementary Table S2: 1040 negative DDI.
